# RORα, a Potential Tumor Suppressor and Therapeutic Target of Breast Cancer

**DOI:** 10.3390/ijms131215755

**Published:** 2012-11-26

**Authors:** Jun Du, Ren Xu

**Affiliations:** 1 Markey Cancer Center, University of Kentucky, Lexington, KY 40536, USA; E-Mail: jdu228@uky.edu; 2 Department of Molecular and Biomedical Pharmacology, University of Kentucky, Lexington, KY 40536, USA

**Keywords:** RORα, tumor suppressor, therapeutic target, breast cancer

## Abstract

The function of the nuclear receptor (NR) in breast cancer progression has been investigated for decades. The majority of the nuclear receptors have well characterized natural ligands, but a few of them are orphan receptors for which no ligand has been identified. RORα, one member of the retinoid orphan nuclear receptor (ROR) subfamily of orphan receptors, regulates various cellular and pathological activities. RORα is commonly down-regulated and/or hypoactivated in breast cancer compared to normal mammary tissue. Expression of RORα suppresses malignant phenotypes in breast cancer cells, *in vitro* and *in vivo*. Activity of RORα can be categorized into the canonical and non-canonical nuclear receptor pathways, which in turn regulate various breast cancer cellular function, including cell proliferation, apoptosis and invasion. This information suggests that RORα is a potent tumor suppressor and a potential therapeutic target for breast cancer.

## 1. Introduction

Inactivation of tumor suppressors is essential for cancer development and progression. It has been shown that a wide variety of tumor suppressors, such as P53 [1], PTEN [2] and some microRNA [3], have the potential to be used as therapeutic targets. Breast cancer is one of the most common malignancies of women worldwide. In 2010, global incidence of breast cancer was about 1,643,000 cases and breast cancer-related women deaths were about 425,000 [4]. Therefore, there is urgent need to identify novel therapeutic targets to fight this mortal disease. We and others recently showed that the orphan nuclear receptor RORα is downregulated in cancer tissues and cell lines and that expression of RORα results in tumor suppressive activities [5–7], suggesting that RORα is a potential drug target for breast cancer treatment.

Aberrant activation of nuclear receptors (NR) during breast cancer progression was observed many years ago. The clinical value of NR as a therapeutic target has already been demonstrated. For example, estrogen receptor-α (ERα), overexpressed in ~70% of breast cancers, is an effective target for the treatment of breast cancer [8]. In contrast, most breast cancers show a down-regulation of retinoic acid receptor (RAR) expression [9], while activation of RAR in breast cancer cells appears to have growth-inhibitory activity [10]. These findings raise hope that perhaps NR may provide new options to prevent progression in human breast cancer.

There are 48 members in the human NR superfamily, which includes receptors for thyroid hormone, steroid hormones, various lipids and oxysterols. The majority of nuclear receptors have well characterized natural ligands, but a few of them are orphan receptors for which no ligand has been identified [11]. Retinoid orphan nuclear receptor (ROR), a subfamily of the orphan nuclear factor family, is so-named because of sequence similarities to the retinoic acid receptor (RAR) and the retinoid X receptor (RXR) [12,13]. In the early 1990s, RORα was identified as the first member of ROR subfamily of orphan receptors. Expression of RORα was found in multiple tissues and cells, including brain, muscle, colon, heart, skin, lung, spleen, leukocytes and mammary epithelial cells [14,15]. Aberrant activation of RORα influences various cellular pathologies, such as osteoporosis, autoimmune diseases, asthma and obesity [16–19]. Furthermore, reduced expression and hypoactivation of RORα in several human tumors, combined with their functional role as tumor suppressors, make RORα an attractive target for cancer therapy.

## 2. RORα Structure

**Figure 1:**
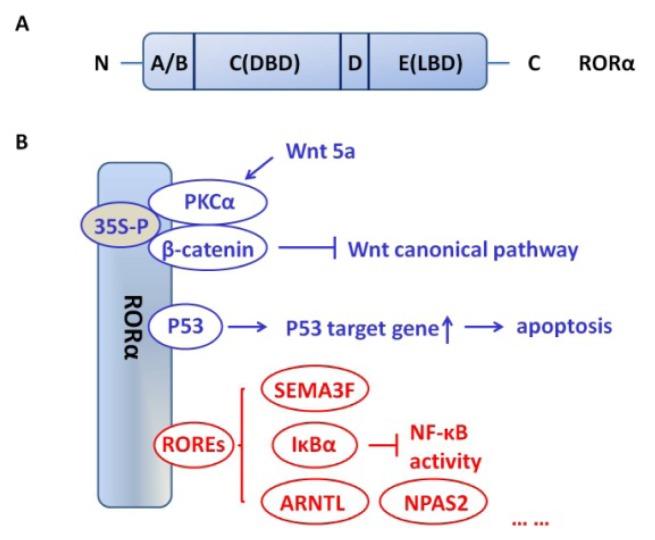
Schematic structure of RORα and interaction of RORα with other proteins andpathways. (**A**) RORα shows a typical domain structure with four major functional domains. The A/B region refers to amino-terminal of RORα. The C region, highly conserved among the ROR family members, is the DNA binding domain (DBD). D is the hinge domain and links the C region to the E region. The E region is the ligand binding domain (LBD); (**B**) Canonical (red) and non-canonical (blue) nuclear receptor activities that may contribute to tumor suppressor function of RORα.

RORα shows a domain structure similar to other NRs with four major functional domains (Figure 1A). The A/B region refers to amino-terminal of RORα. The C region, highly conserved among the ROR family members, is the DNA binding domain (DBD). A relatively short region, D, or the hinge domain, links the C region to the E region. The E region is the ligand binding domain (LBD); in addition to ligand recognition and binding, the LBD also regulates ligand-dependent transcriptional activity. The F region, a carboxy-terminal to the LBD, exists in some NRs [20]. There are four human RORα isoforms, referred to as ROR (α1–α4), while only two isoforms, α1 and α4, have been identified for mice [11]. Isoforms of RORα vary in their A/B domains and display different DNA recognition and transactivation features [13]. Crystallographic studies of RORα suggest that sterols, such as cholesterol, cholesterol sulfate and 7-dehydrocholesterol, may act as a natural ligand of this receptor [21,22]. Recent research has demonstrated that, in human endometrial cells, cholesterol sulfate can regulate expression of the RORα responsive gene NR1D1 without binding to the RORα receptor itself, suggesting that cholesterol sulfate may regulate RORα responsive gene expression, not as a ligand for RORα [23].

## 3. RORα Function in Human Breast Cancer

The *RORα* gene, which is comprised of 15 exons, covers a relatively large 730 kb genomic region. It maps to the middle of chromosome 15q22.2, a region that is highly unstable with frequent breaks and gene rearrangements [24]. Microarray data showed that mRNA levels of RORα are significantly reduced in many cancers (Table 1). RORα has also been identified as one of the methylation-silenced genes in gastric cancer cell lines [25], which favors the concept that reduced RORα expression promotes cancer progression. Downregulation of RORα phosphorylation was observed in colon cancer [26]. While RORα mRNA has been detected in both ER-positive and ER-negative human breast cancer cells [27], the *RORα* gene appears to be down-regulated in breast cancer compared to normal mammary tissue [24,28]. These results suggest that deregulation of RORα contributes to the development of breast cancer.

RORα plays an important role in suppressing malignant phenotypes in culture and *in vivo*. Recently, we reported that inhibition of RORα expression was associated with disruption of polarized acinar structure, the normal cytoarchitecture for breast tissue. Restoration of RORα expression in breast cancer cells resulted in morphologic characteristics associated with less aggressive tumor types: non-branched round spheroid structures in 3D culture, with a colony size and invasive capacity that was significantly reduced [5]. Since disruption of polarized acinar structure is an important early event for breast cancer development, this study suggested that reduced RORα expression contributes to the earliest stages of breast cancer development. In addition, expression of RORα in the mammary epithelial cell line MCF12F significantly inhibited cell proliferation [24]. Activation of RORα in prostate cancer cells affected cell cycle distribution, inducing a decrease in the S phase and a significant decrease of cell proliferation [7]. A recently study showed that introduction of RORα led to an increase of Dox-induced apoptosis in HCT-116 p53+/+ colon cancer cells [6]. Together, these results indicate that RORα is a potent tumor suppressor.

## 4. Potential Pathways that Mediate the Tumor Suppressive Activities of RORα

### 4.1. Canonical *versus* Non-Canonical Pathways

RORα activates nuclear receptor pathways in cancer cells that can be categorized as canonical and non-canonical (Figure 1B). Through these pathways, RORα regulates a variety of cellular activities, such as proliferation, invasion and cell polarization. The canonical RORα pathway involves binding of RORα to ROR response elements (ROREs). ROREs are the specific DNA sequences, AT-rich consensus motifs, in the regulatory region of the target gene [13]. Binding of RORα to the RORE modulates gene transcription and ultimately results in a change in the amount of protein produced. The most distinctive difference between the canonical and non-canonical pathways is the ability of the non-canonical pathway to influence gene expression without binding to ROREs. The mechanism by which RORα influences gene transcription is post-translational modifications and interaction. The significance of this pathway has been emphasized in recent studies.

### 4.2. Role of SEMA3F

SEMA3F is a tumor-suppressive microenvironmental factor that is often inactivated in metastatic cancer [29,30]. This factor has recently been characterized as a RORα-targeted gene [5]. Expression of RORα in breast cancer cells significantly induces SEMA3F transcription and inhibits the mammary tumor invasion in 3D culture [5]. RORE have been identified in the promoter region of the *SEMA3F* gene. Deletion of the RORE in the SEMA3F promoter significantly reduced the transcriptional activation driven by the SEMA3F promoter, indicating that RORα regulates transcription of SEMA3F through canonical nuclear receptor pathways. Moreover, silencing SEMA3F expression in RORα-expressing breast cancer cells rescues the invasive phenotypes in 3D culture, suggesting that tumor suppressor function of RORα is at least partially conferred by SEMA3F. On the other hand, reducing SEMA3F expression has little effect on tumor growth, suggesting that the tumor suppressor function of RORα involves other target genes and pathways as well [5].

### 4.3. Role of Wnt/β-Catenin

RORα activity is regulated by various post-translational modifications, including phosphorylation, ubiquitination and SUMOylation. Lee and colleagues showed that Wnt5a/PKC induces phosphorylation of RORα on serine residue 35 [26]. Wnt signaling can use the canonical (β-catenin dependent) and non-canonical (β-catenin independent) pathways. The canonical Wnt signaling pathway has been implicated in supporting breast transformation to cancer and in tumor progression [31,32]. Wnt5a activates non-canonical Wnt signaling and directs a breast cancer-suppressing effect [33,34]. Phosphorylated RORα, induced by Wnt5a/PKC pathway activation, attenuates the canonical Wnt signaling pathway. The inhibition is accomplished through binding of RORα to β-catenin, which suppresses the transcription of Wnt/β-catenin target genes. The transrepression mechanism of RORα on β-catenin is achieved, at least in part, by competition with a subset of coactivators for β-catenin binding and, possibly, recruitment of histone lysine methyltransferases, which results in transcriptional repression [26]. Therefore, RORα may suppress breast cancer progression by inhibiting Wnt/β-catenin target genes.

### 4.4. Role of p53

It is well-established that p53-regulated apoptosis and DNA repair are important in preventing cancers and that aberrant p53 function promotes breast cancer development and progression [35,36]. RORα has recently been identified as a direct p53 target gene. DNA damaging agents, such as doxorubicin and ionizing radiation, induce RORα expression in a p53-dependent manner [6]. Interestingly, RORα can also enhance DNA damage-induced apoptosis through p53 in colon cancer cells. It is revealed by genome-wide analysis that RORα could regulate p53-responsive genes, which mainly influence apoptosis. Further study also showed that RORα regulates p53 stability and p53 transcription activation in a HAUSP/Usp7-dependent manner [6]. Although enhancing p53 target gene by RORα is also reported in hepatocellular carcinoma cells [37], it remains to be determined whether RORα could stimulate breast cancer cell apoptosis via such an interaction with p53.

### 4.5. Role of Hypoxia/Angiogenesis

Clinical evidence showed that hypoxia is associated with angiogenesis and a poor prognosis in patients with invasive breast cancer [38]. Other *in vivo* studies demonstrated that ischemia-induced angiogenesis was enhanced in RORα-deficient mice. RORα (sg/sg) mice had an increased angiogenic score and capillary density within the ischemic hindlimb, suggesting that RORα is a potential inhibitor of angiogenesis. In addition, more extensive angiogenesis correlated with an increased expression of endothelial nitric oxide synthetase (eNOS ) protein, whereas the level of the anti-angiogenic cytokine IL-12 was significantly reduced [39]. These observations suggest that RORα may participate in the control of gene transcription in response to hypoxic stress and functions as an important negative modulator of angiogenesis in breast cancer. HIF-1α is involved in tumor angiogenesis and metastasis by regulating genes involved in response to hypoxia [40]. Transcriptional activation of RORα4, but not RORα1, is induced under hypoxic conditions by HIF-1α in human hepatoma cells [41,42]. These studies suggest that RORα may be a potential target of hypoxic stress and is involved in the regulation of angiogenesis.

### 4.6. Role of NF-κβ

Emerging evidence demonstrates that RORα is a crucial regulator of the NF-κB pathway [43,44]. Ectopic expression of RORα in human primary smooth-muscle cells inhibits NF-κB-dependent promoter activity and NF-κB-responsive genes, such as *IL-6*, *IL-8* and *COX-2*. Further analysis showed that RORα negatively interferes with the NF-κB signaling pathway by activating IκBα transcription [44]. In addition, it has been shown that NF-κB-responsive genes IL-6 and COX-2 can be up-regulated to Rev-ERBα [45], while the activity of Rev-ERBα can be competitively inhibited by RORα [46]. Transcription factor NF-κB regulates a variety of cancer related processes, including immune-response, cell survival and cancer invasion [47]. Elevated NF-κB binding activity has been observed in both breast cancer cell lines and primary human breast cancer tissues and contributes to the activation of cell-cycle related genes and various microenvironmental cues [48–50]. Thus, it is worthwhile to explore whether the RORα suppresses breast cancer progression through inhibition of the NF-κB signaling pathway.

### 4.7. Role of Circadian-Related Genes

Disruption of circadian rhythms is associated with an elevated risk of breast cancer [51,52]. It has been demonstrated that SNPs of NPAS2 and downregulation of PERs correlates with breast cancer development and progression [53,54]. Furthermore, PER2 deficient mice are prone to develop cancer in response to radiation [55]. These results suggest that aberrant activation of circadian genes contributes to breast cancer development. RORα-deficient mice exhibit aberrant circadian behavior, indicating that RORα is a potent regulator of circadian rhythms. It has been shown that RORα regulates Bmal1 expression and consolidates daily locomotor activity in the suprachiasmatic nucleus [56]. Moreover, RORE has been identified in the promoter regions of BMAL1 and NPAS2 [57,58], indicating that the RORα regulates circadian genes expression through the canonical pathway. However, it remains to be determined whether RORα modulates circadian rhythms in breast cancer cells and how disruption of circadian rhythms promote breast cancer progression.

### 4.8. Interaction with Other NR

Cross-talk with or modulation of other nuclear receptors, such as estrogen receptor (ER), is another important function of RORα. It has been shown that RORα cooperates with ER to induce cyclin D1 expression in the ER-positive breast cancer cell line MCF-7 [59]. RORα also significantly augmented the expression and activity of aromatase (an enzyme complex that catalyzes the conversion of androgens to estrogens) in MCF-7 cells [60]. Although RORα appears to be a potential ERα partner, RORα seems to be expressed differently than ER in breast cancer cells; no correlation was found between RORα expression and ERα status [61]. Interestingly, we found that RORα imparts some cancer-suppressive activities in the ER-negative breast cancer cell lines MDA-MB-231, MDA-MB-157 and T4-2, such as inhibition of cell migration and proliferation. *In vivo*, tumors formed by RORα-expressing MDA-MB-231 cells were also much smaller than tumors formed from the wild-type cells [5]. But, the same treatment has little effect on ER positive cell lines (data not shown). Thus RORα may have different activity in ER-positive and -negative breast cancer cells, and the mechanism whereby RORα differentially regulates cellular response in ER-positive and -negative cells remains to be elucidated.

It is most likely that tumor suppressor function of RORα is mediated by multiple pathways and involves canonical and non-canonical nuclear receptor activity. In addition, crosstalk among those pathways has been observed *in vitro* and *in vivo*; therefore, an integrated view of RORα downstream signaling is crucial for our understanding of roles of this protein in breast cancer progression.

## 5. ROR α as a Drug Target

RORα-targeted therapeutics may efficiently suppress certain types of tumors, thus it is crucial to identify potent ligands or agonists that have the potential to be used in cancer treatment. In fact, a recent pharmacokinetic study indicates that SR1078, a synthetic agonist for the orphan nuclear receptors RORα and RORγ, induces expression of two ROR target genes, glucose-6-phosphatase and FGF21 in mice [62]. Treatment with SR1078 enhances apoptosis of liver cancer cells in culture, suggesting that the RORα agonist may be a potent inhibitor of cancer progression [37]. In addition, melatonin, secreted by the pineal gland, has been suggested as the natural ligand for RORα [63,64]. Increasing evidence suggests that melatonin has the potential be used in breast cancer prevention and therapeutically [52,65]. Melatonin treatment induced apoptosis in the murine colonic cancer; the effect was diminished by RZR/RORα antagonist CGP 55644 [66,67]. Thus, it is important to explore whether the RORα plays a key role in melatonin-mediated inhibition of cell invasion and proliferation of breast cancer cells. Hopefully, RORα-specific, clinically-useful agonists for breast cancer treatment will be identified and tested in the future.

## 6. Conclusions

The orphan nuclear receptor RORα has recently been identified as a potent tumor suppressor [5,7,26,67]. Expression of RORα is downregulated in breast cancer tissues and cell lines. Restoration of RORα expression in cancer cells suppresses the malignant phenotypes in culture and *in vivo*[5]. Based on these observations and given the recent progress characterizing RORα agonists, further investigations of tumor suppressor activities by RORα in breast cancers may lead to the discovery of novel therapeutic targets for this mortal disease.

## Figures and Tables

**Table 1:** Analyzing published microarray datasets show that the mRNA levels of RORα is downregulated in various cancers; numbers in the table show how many datasets passed the threshold (cancer *vs*. normal: 1.5 fold change and *p* < 0.05). Blue represents the datasets in which the mRNA levels of RORα are downregulated in cancer tissues compared to normal tissues, while the datasets with upregulated RORα in cancer tissue are shown in red.

Analysis type by cancer	Normal *vs.* Cancer	
bladder cancer	1	
brain and CNS cancer		2
breast cancer	9	2
cervical cancer	3	
colorectal cancer	5	
esophageal cancer	7	
gastric cancer	2	
head and neck cancer	5	
kidney cancer	1	1
leukemia	9	2
liver cancer	1	
lung cancer	2	1
lymphoma	4	4
melanoma	3	1
myeloma	1	1
other cancer	9	
ovarian cancer	1	
pancreatic cancer	2	
prostate cancer	2	
sarcoma	1	1
significant unique analyses	68	15
total unique analyses	381	
